# Novel anatomical landmark guided puncture method in L4/L5 spine posterior interlaminar endoscopic surgery: a technical note and case series

**DOI:** 10.3389/fsurg.2025.1715171

**Published:** 2025-12-15

**Authors:** Bin Zheng, Tiepeng Han, Yan Liang, Haiying Liu, Panfeng Yu

**Affiliations:** Spine Surgery, Peking University People’s Hospital, Beijing, China

**Keywords:** interlaminar approach, fluoroscopic guidance, puncture positioning, anatomical landmark, radiation dose

## Abstract

**Background:**

L4/L5 segment lumbar disc herniation and canal stenosis commonly cause low back and leg pain. Posterior interlaminar spine endoscopy has proven efficacy, but puncture positioning relies on experience and requires multiple fluoroscopic exposures, increasing operative difficulty and radiation exposure. This study proposes a vertebral anatomy-based puncture point(Yu Landmark) to assist puncture operations.

**Methods:**

A retrospective analysis of 426 L4/L5 posterior interlaminar spine endoscopy patients is conducted, divided into Yu landmark group (205 cases) and conventional group (221 cases). The Yu landmark determines the puncture entry point through the intersection of two lines under anteroposterior fluoroscopy: a vertical line from the midpoint of the L4 inferior articular process and a tangent line from the highest point of the junction between the L4 spinous process base and L4 lamina inferior edge. Puncture efficiency, fluoroscopic usage, complications, and clinical outcomes are compared between groups.

**Results:**

The Yu landmark group shows significantly reduced fluoroscopic exposures (4.9 ± 1.4 vs. 22.7 ± 4.8), radiation dose (0.48 ± 0.23 vs. 1.34 ± 0.29 mGy), and channel establishment time (22.6 ± 4.7 vs. 29.6 ± 5.9 min) (all *P* < 0.01), with higher single-puncture success rate (95.1% vs. 82.4%, *P* < 0.01). Early postoperative VAS and ODI improvements are better, while long-term outcomes and complications are similar.

**Conclusions:**

The Yu landmark is simple, objective, and reproducible, significantly reducing fluoroscopic exposure while improving puncture efficiency, providing a standardized positioning strategy for L4/L5 posterior interlaminar endoscopic surgery.

## Introduction

L4/L5 segment lumbar disc herniation and spinal stenosis are common causes of lower back pain and leg pain. Posterior interlaminar spine endoscopy has become a widely used minimally invasive approach for treating these conditions, with advantages of minimal muscle trauma and rapid recovery (1). The critical step for posterior interlaminar spine endoscopy success is precise and safe percutaneous puncture to establish a working channel into the target area (2).

Traditionally, surgeons rely on experience and biplanar fluoroscopic guidance, repeatedly adjusting needle trajectory until the needle tip reaches the optimal target position. This “trial-and-error” freehand technique is challenging for beginners, as accurate placement of the working cannula often requires multiple attempts and frequent imaging verification. Studies show that conventional fluoroscopy-guided posterior interlaminar spine endoscopy typically requires multiple needle insertions (averaging over 5 attempts) and dozens of x-ray fluoroscopic images per case, particularly for less experienced surgeons (3, 4).

This prolonged fluoroscopic guidance not only extends operative time but also increases radiation exposure for patients and surgical personnel (5, 6). Excessive fluoroscopy and repeated puncturesare associated with decreased surgeon confidence, increased risk of nerve root irritation, and greater cumulative radiation dose. Therefore, reducing the number of puncture attempts and fluoroscopic usage is an ideal goal for improving surgical efficiency and safety.

Inspired by the principle of using anatomical landmarks for guidance, we develop a new puncture point positioning method specifically for L4/L5 posterior approach surgery, called the “Yu landmark”. This technique utilizes visible bony landmarks under fluoroscopy to define the optimal skin entry point, aiming to make the initial needle trajectory as close as possible to the ideal path for entering the L4/L5 intervertebral area. The Yu landmark determines the puncture entry point through the intersection of two lines under anteroposterior fluoroscopy: a vertical line from the midpoint of the L4 inferior articular process and a tangent line from the highest point of the junction between the L4 spinous process base and L4 lamina inferior edge. The intersection of these two lines on the patient's skin marks the Yu landmark puncture entry point. At this point, the needle is inserted perpendicular to the skin toward the L4/L5 intervertebral disc.

This study aims to evaluate Yu landmark vs. traditional experience-based puncture methods in puncture efficiency, fluoroscopic exposure, first-pass success rate, and clinical outcomes in L4/L5 interlaminar endoscopic surgery.

## Methods

### Study design and patient selection

We conduct a single-center retrospective controlled study including patients who undergo L4/L5 posterior interlaminar endoscopic surgery at our institution in recent years. After screening according to strict inclusion and exclusion criteria, a total of 426 patients are included (detailed criteria below). All patients have single-segment L4/L5 disc herniation or spinal stenosis and undergo IELD or LE-ULBD via posterior interlaminar endoscopic approach.

Patients are divided into two groups based on the puncture positioning technique used during surgery:

1. **Yu landmark group (anatomical landmark positioning):** 205 cases using the new landmark-guided puncture point method
2. **Traditional method group (conventional freehand positioning):** 221 cases where surgeons use standard experience-based freehand methods to select puncture points under fluoroscopy

All surgeries are performed by highly experienced spinal endoscopic surgeons proficient in POSTERIOR INTERLAMINAR SPINE ENDOSCOPY to minimize variability due to surgeon skill or learning curve changes over time. We retrospectively review medical records, operative reports, and imaging studies, extracting patient demographics, disease characteristics, surgical details, and outcomes. The institutional ethics committee approves the study protocol, and all patients provide consents.

### Inclusion and exclusion criteria

#### Inclusion criteria

1. **L4/L5 disc herniation or spinal stenosis:** Diagnosis of symptomatic lumbar disc herniation or L4/L5 spinal stenosis at the L4/L5 segment, with MRI/CT showing corresponding L4 or L5 nerve root compression, persistent radicular pain, or neurogenic claudication.
2. **Conservative treatment failure:** Symptoms ineffective to adequate conservative treatment (such as physical therapy, medications) for at least 6–12 weeks, thus requiring surgical intervention.
3. **Age ≥18 years:** Adults without contraindications to minimally invasive surgery, able to tolerate prone anesthetic positioning.

#### Exclusion criteria

1. **Multi-segment or non-L4/L5 pathology:** Cases requiring multi-segment surgery or disease at segments other than L4/L5 in the same operation.
2. **Severe segmental degeneration or deformity:** Cases with severe anatomical abnormalities at L4/L5, such as advanced Modic endplate changes, near-complete disc height collapse, large osteophytes, or congenital anomalies (such as L5 sacralization, hemivertebra, congenital spinal stenosis), which significantly distort normal anatomy and potentially alter bony landmarks.
3. **Spinal instability or deformity:** Dynamic instability (such as L4/L5 grade II or higher spondylolisthesis) or significant spinal scoliosis/kyphosis beyond the indications for MIS endoscopic treatment.
4. **Previous L4/L5 surgical history:** History of L4/L5 open surgery or fusion, or any condition that alters normal anatomical landmarks (scar tissue, surgical fusion mass).
5. **Medical contraindications:** Severe medical comorbidities excluding surgery or anesthesia (uncontrolled heart disease, coagulopathy, active infection, etc.), or active systemic infection or malignancy.
6. **Other contraindications:** Patients unable to cooperate or follow up (such as severe psychiatric illness), or any other circumstances deemed by the surgical team to make endoscopic surgery unsuitable.

### Puncture positioning techniques

#### Yu landmark group

**Figure 1:**
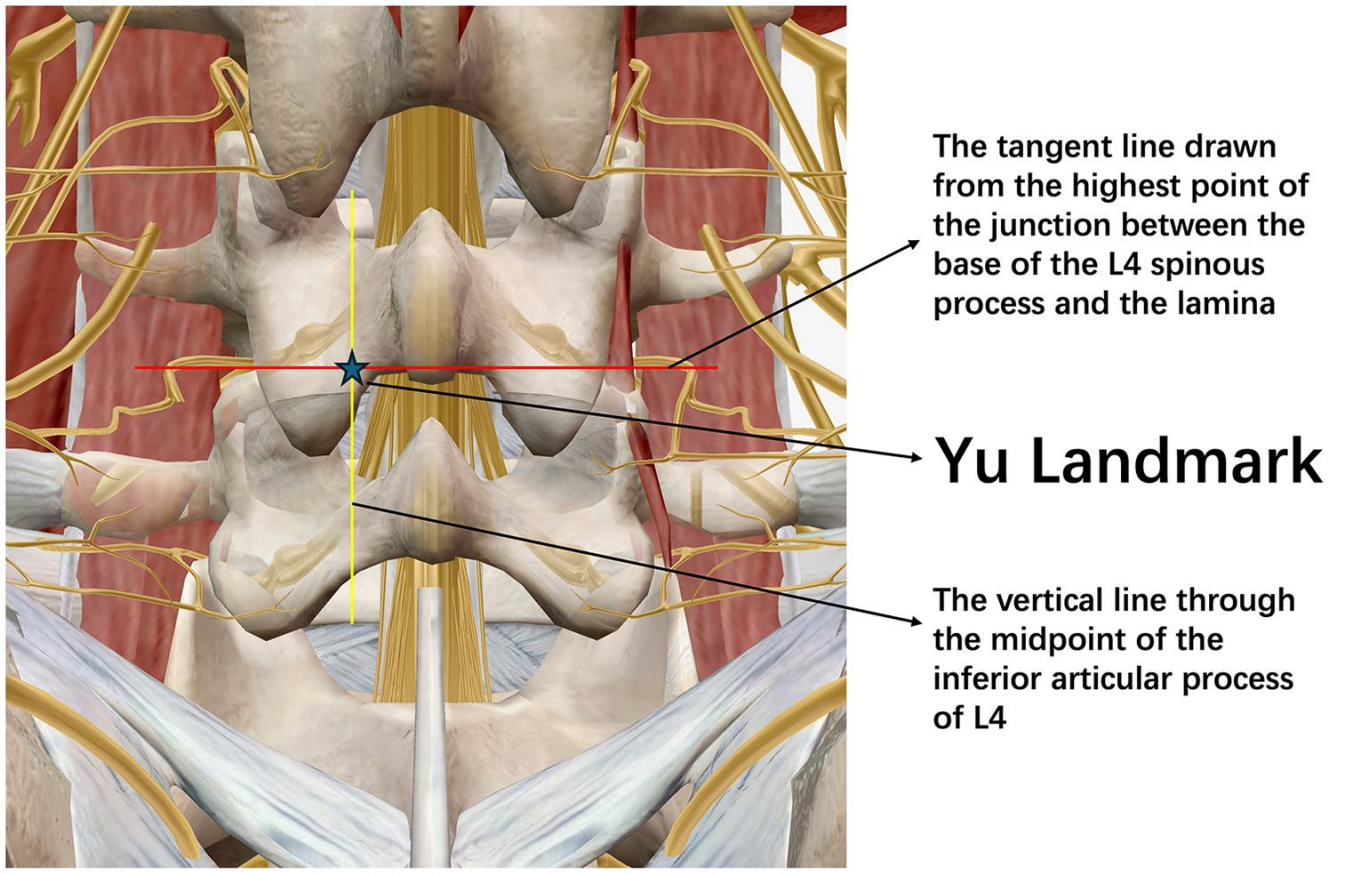
Posterior anatomical illustration of L4/L5 segment: definition of Yu landmark. The Yu landmark (indicated by asterisk) is determined by the intersection of two key bony anatomical reference lines: a vertical line passing through the midpoint of the L4 inferior articular process, and a horizontal line tangent to the highest point of the junction between the L4 spinous process base and lamina. The intersection of these two lines represents the optimal entry point for L4/L5 interlaminar approach needle insertion, providing a stable anatomical basis for puncture in posterior interlaminar spine endoscopy.

**Figure 2:**
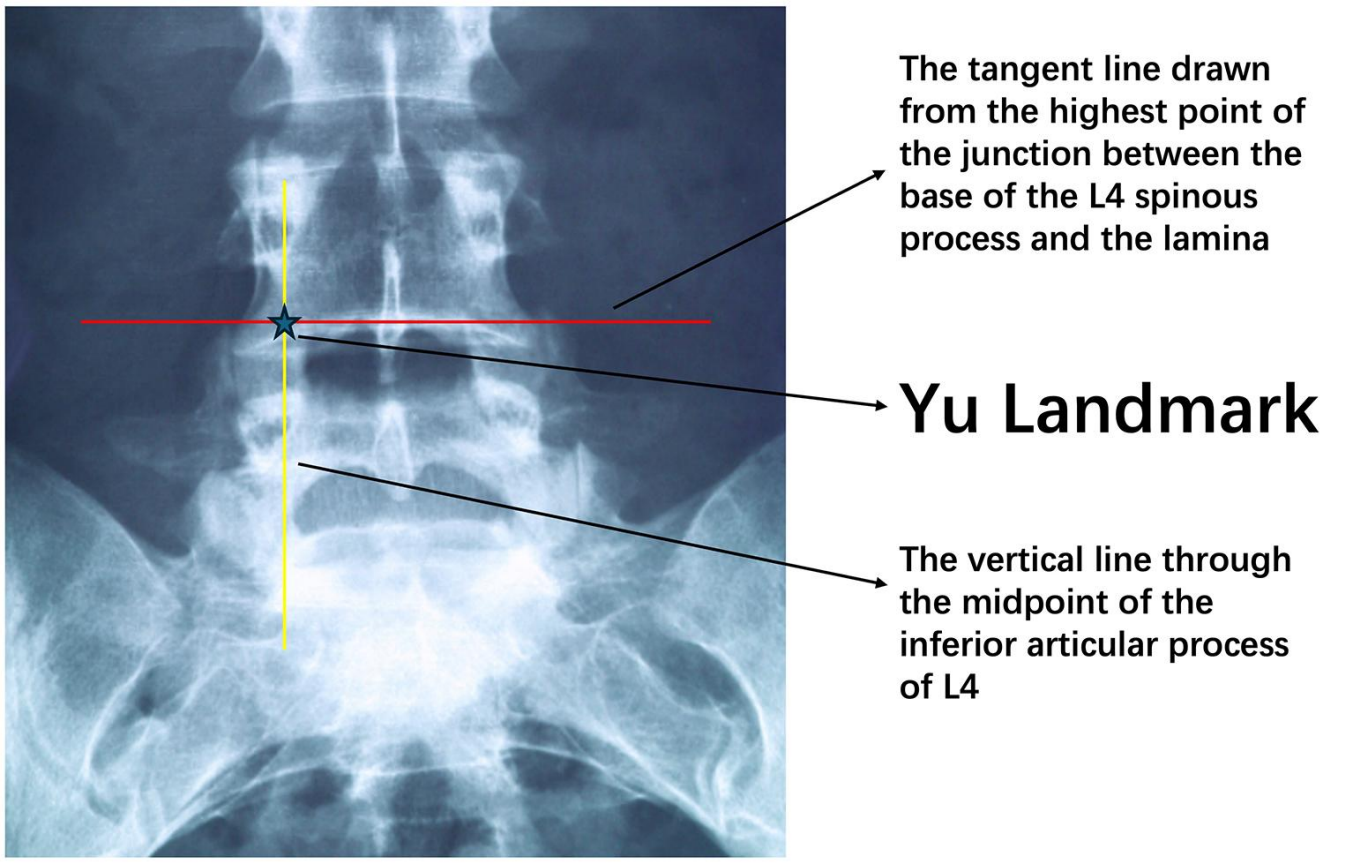
Intraoperative fluoroscopic validation of the Yu landmark concept. Anteroposterior x-ray imaging of the L4/L5 region demonstrates the same anatomical reference lines as shown in Figure 1. The vertical line passes through the midpoint of the L4 inferior articular process, and the horizontal line is tangent to the highest point of the junction between the L4 spinous process base and lamina. The convergence point of these two lines (indicated by asterisk) represents the Yu landmark, indicating the ideal puncture position. This intraoperative fluoroscopic image confirms that the Yu landmark is easily identifiable within the patient's body and provides reliable localization guidance for surgeons.

After routine sterile draping with the patient in the prone position, the surgeon precisely localizes the L4/5 level under C-arm fluoroscopy. On a true anteroposterior (AP) view, the midpoint of the L4 inferior articular process on the affected side is identified and a vertical line parallel to the spinal axis is projected onto the skin. The highest point at the junction between the L4 spinous process base and the inferior edge of the L4 lamina is then identified, and a tangent line along the posterior laminar edge is marked on the skin. The intersection of these two lines on the skin is defined as the Yu landmark, i.e., the posterior interlaminar entry point (Figures 1, 2).

An 18-G spinal needle is introduced perpendicular to the skin at the Yu landmark. On AP, the trajectory remains lateral to the midline and is directed toward the medial border region of the midpoint of the L4 inferior articular process; the needle should not cross the midline before bony contact and corridor creation. On lateral, the tip advances along the posterior laminar plane toward the L4/5 posterior interlaminar window, with depth titrated to the posterior third of the interlaminar space. When the predetermined target is reached (bony contact at the inner laminar edge/ligamentum flavum junction), the stylet is removed and a soft guidewire is inserted.

The posterior interlaminar working corridor is then established over the guidewire using sequential dilators and limited bony work along the inner edge of the lamina and the medial margin of the L4 inferior articular process (partial undercutting facetectomy as needed), while preserving ≥50% of the facet. A working cannula is placed. AP fluoroscopy confirms the cannula at the lateral border of the spinal canal facing the dural sac, and lateral fluoroscopy confirms appropriate depth at the posterior third of the interlaminar space. The endoscope is connected to complete procedures such as nucleus pulposus removal and decompression.

#### Traditional method group

With the patient in the same prone position, after localizing the L4/L5 interspace under C-arm fluoroscopy, the surgeon estimates the needle entry point based on preoperative imaging judgment and surface palpation experience. Usually, a puncture skin incision is made on the affected side at the level slightly above the iliac crest, approximately 10–12 cm from the posterior midline.

After local anesthesia, the surgeon inserts the needle at an empirically set angle (generally obliquely toward the cranial side with an inward inclination of approximately 30°–45° for the L4/L5 segment). During needle insertion, intermittent fluoroscopy is performed, observing the needle tip position relative to the pedicle and intervertebral space on AP view, and needle tip depth approaching the posterior third of the intervertebral disc on lateral view.

Based on fluoroscopic feedback, if the initial needle insertion does not reach a satisfactory position, the needle tip is partially or completely withdrawn, and the needle insertion angle or position is adjusted before puncturing again. This process is repeated empirically until the needle tip reaches the ideal foraminotomy area—near the posterosuperior edge of the lower vertebral body on lateral view, and near the medial edge projection of the pedicle on AP view.

Subsequent guidewire placement, channel dilation, and endoscopic removal of herniated nucleus pulposus or LE-ULBD steps are the same as in the new method group.

### Surgery-related outcome measures

We evaluate multiple outcome indicators to compare the efficiency, safety, and effectiveness of the new puncture positioning method vs. the traditional method:

1. **Puncture channel establishment time (minutes):** Time from initial needle insertion to successful placement of the working cannula at the target position.
2. **Number of fluoroscopic exposures:** Total number of x-ray fluoroscopic images taken during the puncture and working channel establishment phases. This is an objective indicator of how much intraoperative imaging is needed to position the needle and verify placement. Fewer exposures indicate more efficient and confident positioning.
3. **Fluoroscopic radiation dose (mGy):** Cumulative radiation dose recorded by the C-arm during surgery (we use dose-area product values converted to mGy equivalent patient dose).
4. **Single puncture success rate:** Cases where the initial puncture (first needle insertion) successfully reaches the target without requiring needle withdrawal and redirection.
5. **Puncture-related intraoperative complications:** We record any complications occurring during puncture or dilation, such as transient nerve root irritation (e.g., radiating limb pain or paresthesias when the needle or dilator contacts nerve roots), dural tears (cerebrospinal fluid leak), vascular injury, or other malpositioning events (e.g., needle accidentally entering the spinal canal or abdominal cavity). The incidence of these events is recorded for each group.
6. **Surgical indicators:** Total operative time, type of surgery (IELD, LE-ULBD).

### Clinical efficacy indicators

1. **Postoperative pain relief (VAS scores):** Low back pain and leg pain are assessed using the Visual Analog Scale (VAS, 0 = no pain, 10 = most severe pain). We record VAS scores preoperatively and at several postoperative time points (immediately postoperative or at discharge, 1 month postoperatively, and longer-term follow-up such as 6 or 12 months). VAS improvement is calculated as change from preoperative baseline.
2. **Functional improvement (Oswestry Disability Index, ODI):** ODI is measured preoperatively and postoperatively (such as 1 month and 1 year) (0%–100%, higher scores indicate greater disability) to assess improvement in daily function after surgery. We compare the degree of ODI improvement between the two groups at early and late follow-up.

### Statistical analysis

All data are analyzed using SPSS 26.0 (IBM Corp.). We first perform descriptive statistics for both groups. Continuous variables are expressed as mean ± standard deviation (SD) if approximately normally distributed, or as median and interquartile range if skewed. Categorical variables are expressed as counts.

For continuous variable indicators, we first assess normality and homogeneity of variance. If assumptions are met, independent samples t-tests are used to compare means between groups. If data do not follow normal distribution, we use non-parametric Mann–Whitney U tests to compare group medians.

For binary or categorical outcomes, chi-square (*χ*^2^) tests are applied to assess differences in proportions. All tests are two-sided, and *p*-values <0.05 are considered statistically significant.

## Results

A total of 205 patients in the “anatomical positioning method” (Yu Landmark positioning method) and 221 patients in the “traditional positioning method” are included. All patients complete follow-up, and no patients are lost to follow-up during the study period. The average follow-up is 13.5 ± 1.13 months. The two groups are comparable in preoperative general conditions, with no statistically significant differences in gender ratio (male 108/female 97 vs. male 117/female 104), BMI (21.90 ± 3.13 vs. 21.47 ± 3.39), and age (48.4 ± 10.4 years vs. 49.4 ± 10.4 years). The distribution of surgical methods is comparable between groups (IELD vs. LE-ULBD: 96/109 cases vs. 114/107 cases, *p* = 0.33). Results are summarized in Table 1.

**Table 1:** Baseline characteristics and surgical outcomes of Yu landmark vs. traditional puncture methods

Variable	Anatomical landmark method (Mean ± SD)	Conventional method (Mean ± SD)	*P* value
Sex (Male/Female)	108/97	117/104	0.96
BMI (kg/m^2^)	21.90 ± 3.13	21.47 ± 3.39	0.17
Age (years)	48.4 ± 10.41	49.38 ± 10.43	0.17
Number of fluoroscopies	4.90 ± 1.41	22.69 ± 4.75	<0.01
Radiation dose (mGy)	0.48 ± 0.23	1.34 ± 0.29	<0.01
Channel establishment time (min)	22.62 ± 4.72	29.57 ± 5.87	<0.01
First puncture success	195/10	182/39	<0.01
Surgical method: Discectomy/ULBD	96/109	114/107	0.33
Total operative time (min)	98.65 ± 23.19	98.62 ± 22.92	0.99
Preoperative VAS	5.91 ± 1.47	6.02 ± 1.40	0.45
Postoperative VAS Day 1	2.03 ± 0.81	2.52 ± 1.15	<0.01
Postoperative VAS 1 month	1.43 ± 1.09	1.43 ± 1.17	0.94
Postoperative VAS 1 year	1.00 ± 0.84	1.01 ± 0.85	0.82
Preoperative ODI	52.55 ± 4.91	52.55 ± 4.72	1.00
Postoperative ODI Day 1	34.15 ± 4.77	39.90 ± 5.13	<0.01
Postoperative ODI 1 month	26.77 ± 5.01	27.47 ± 5.48	0.17
Postoperative ODI 1 year	20.14 ± 2.99	20.26 ± 3.20	0.69

### Surgery-related outcome measures

#### Fluoroscopic exposures and dose

The anatomical positioning group has significantly fewer intraoperative fluoroscopic exposures than the traditional group (4.90 ± 1.41 vs. 22.69 ± 4.75, *p* < 0.01, 95% Confidence Interval of Difference −18.47 to −17.11); radiation dose is also significantly reduced (0.48 ± 0.23 vs. 1.34 ± 0.29, *p* < 0.01, 95% Confidence Interval of Difference −0.91 to −0.81). This indicates that the new method significantly reduces the frequency of intraoperative x-ray fluoroscopy and radiation exposure.

#### Channel establishment time and puncture success rate

The anatomical positioning group has shorter working channel establishment time (22.62 ± 4.72 min vs. 29.57 ± 5.87 min, *p* < 0.01, 95% Confidence Interval of Difference −7.96 to −5.92). Additionally, this group has a significantly improved single-puncture success rate of 195/205 cases (95.1%), significantly higher than the traditional group's 182/221 cases (82.4%, *p* < 0.01, OR = 4.18, 95% CI: 2.03–8.62). This indicates that the new positioning method can complete channel placement more rapidly and improve the success rate of hitting the target with the first puncture.

Total operative time is similar between groups (approximately 98.6 ± 23 min vs. 98.6 ± 23 min, *p* = 0.990).

### Clinical outcome results

#### Postoperative VAS improvement

**Figure 3:**
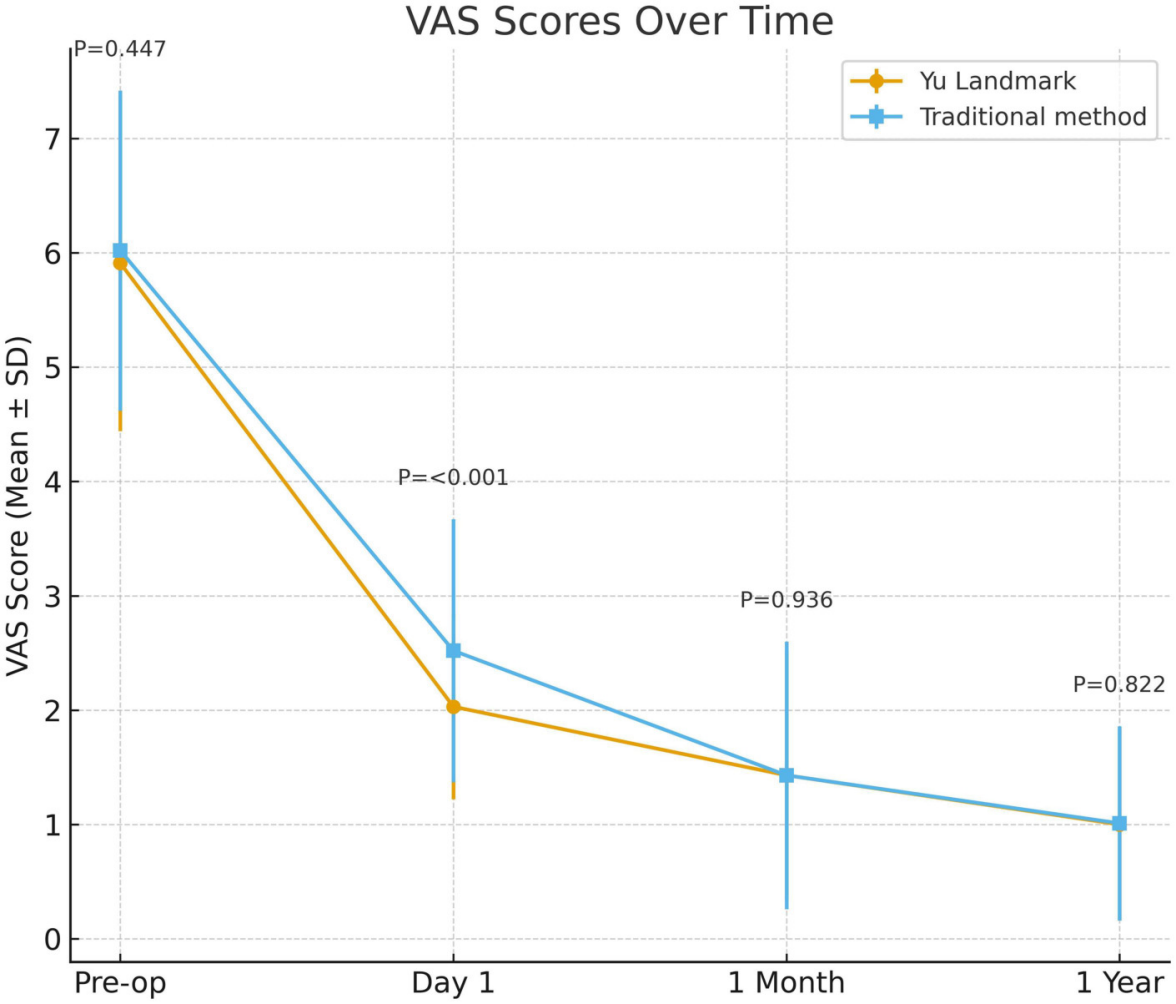
Postoperative pain improvement comparison: Yu landmark group vs. conventional group visual analog scale (VAS). The line graph displays VAS scores for both patient groups at preoperative and various postoperative follow-up time points. Both groups demonstrated similar severe pain preoperatively. At 1 day postoperative, the Yu landmark group showed significantly lower VAS scores compared to the conventional group (*P* < 0.001), indicating superior immediate analgesic efficacy of this method. At 1 month postoperative, both groups achieved low VAS levels with no significant difference (*P* = 0.936). At 1-year follow-up, both groups maintained pain scores at the lowest levels, with no statistically significant difference between groups (*P* = 0.822).

Both groups show significant pain relief compared to preoperative levels. On postoperative day 2, the anatomical positioning group VAS pain score decreases to 2.03 ± 0.81, significantly lower than the traditional group's 2.52 ± 1.15 (*p* < 0.01). At 1 month postoperatively, both groups have VAS scores of approximately 1.43, with no statistically significant difference; at 1 year postoperatively, VAS is maintained at approximately 1 point, with no significant difference between groups. The follow-up is shown in Figure 3.

#### Postoperative ODI improvement

**Figure 4:**
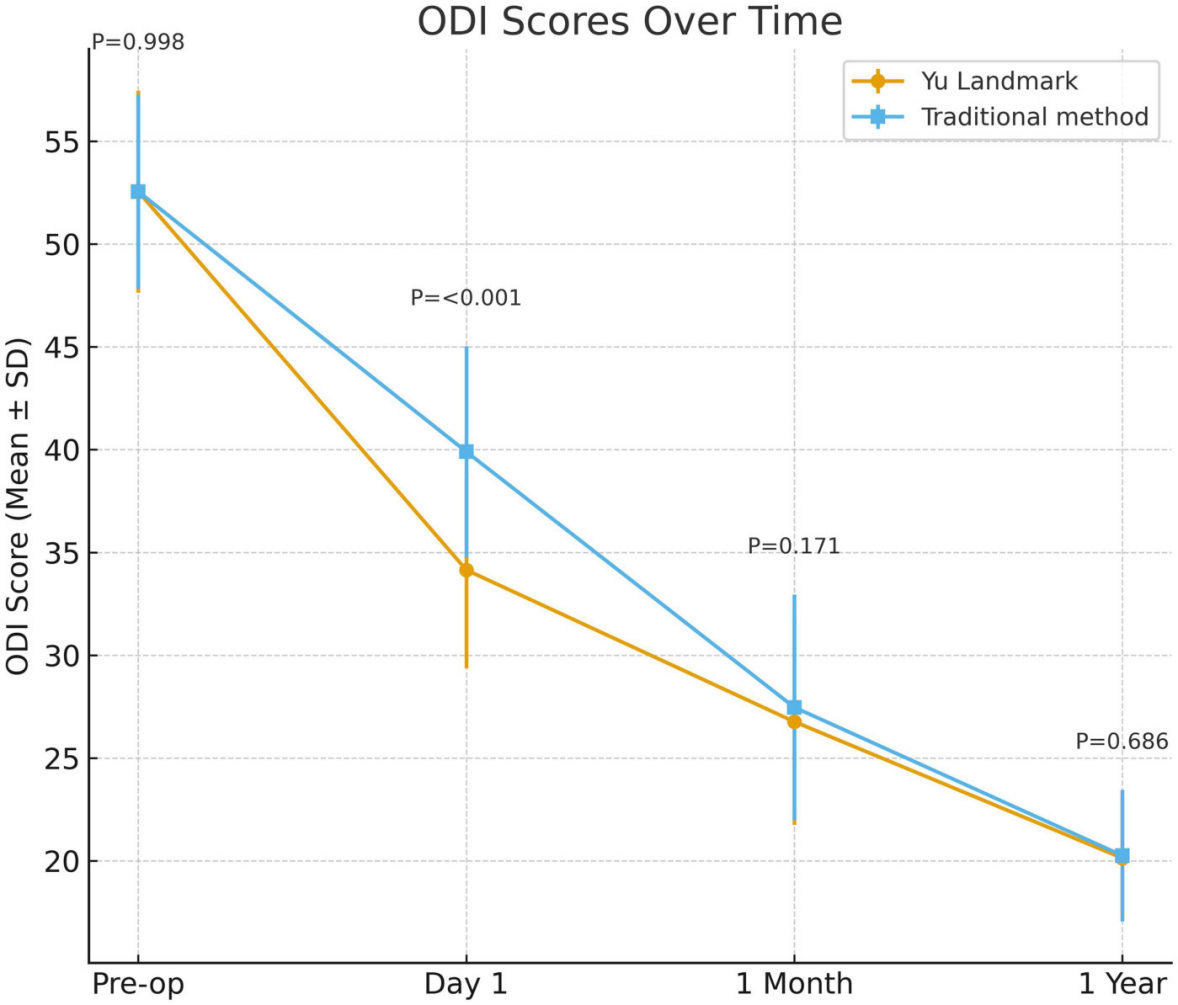
Postoperative functional recovery comparison: Yu landmark point group vs. conventional group Oswestry disability index (ODI). The line graph compares mean ODI (±SD) between the two patient groups at preoperative, 1 day postoperative, 1 month postoperative, and 1 year follow-up time points. At 1 day postoperative, the Yu landmark point group demonstrated significantly lower ODI compared to the conventional group (approximately 35 vs. 42, *P* < 0.001), indicating superior early postoperative functional recovery. At 1 month postoperative, both groups showed marked improvement in ODI and converged to similar levels. At 1-year follow-up, both groups maintained low ODI levels with no statistically significant difference (*P* = 0.686).

Both groups show improvement in Oswestry Disability Index (ODI) compared to preoperative levels. On postoperative day 1, the anatomical positioning group ODI decreases to 34.15 ± 4.77, significantly lower than the traditional group's 39.90 ± 5.13 (*p* < 0.001), indicating better early functional recovery in the new method group. At 1 month postoperatively, both groups' ODI decreases to approximately 27, with no statistically significant difference (*p* = 0.17); at 1 year postoperatively, ODI further decreases to approximately 20, with no significant difference between groups. The follow-up is shown in Figure 4.

#### Other indicators with no significant differences

The above results indicate that the two methods are comparable in terms of total operative time and preoperative pain levels. No cases of dural tear or intraoperative bleeding occurred in either group. Minor transient nerve irritation is occasionally observed but resolved spontaneously without sequelae. One patient in the Yu landmark group and two patients in the traditional group experienced mild nerve injury, with no statistically significant difference between groups (*p* > 0.05).

### Typical case demonstration

**Figure 5:**
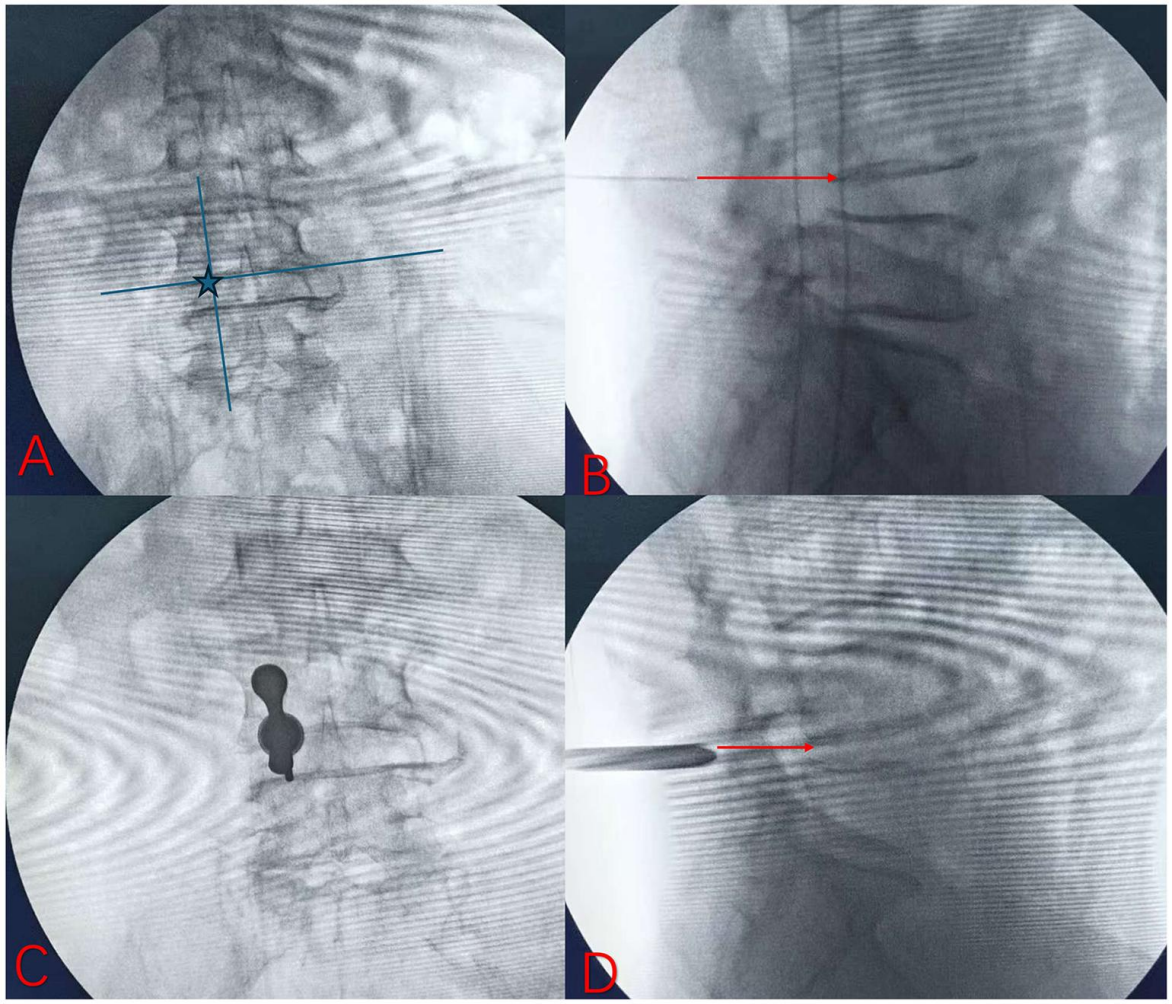
Puncture and channel establishment guided by Yu landmark (intraoperative fluoroscopic images of L4/L5 posterior interlaminar spine endoscopy) **(A)** anteroposterior fluoroscopic view showing the Yu landmark (intersection of anatomical reference lines). **(B)** Puncture needle inserted percutaneously from the Yu landmark, following the planned trajectory toward the intervertebral disc. **(C)** Working channel establishment image under anteroposterior fluoroscopy. **(D)** Working channel establishment image under lateral fluoroscopy.

To further illustrate the clinical application of the Yu landmark, representative intraoperative fluoroscopic images are shown in Figure 5. On the anteroposterior view, the intersection of the anatomical reference lines identified the puncture entry point (Figure 5A). Guided by this landmark, the puncture needle is advanced along the planned trajectory (Figure 5B), followed by sequential dilation and placement of the working channel (Figure 5C). Final confirmation of the working cannula position at the L4/L5 interlaminar space is obtained under fluoroscopy (Figure 5D).

## Discussion

This study evaluates a novel fluoroscopy-guided puncture positioning technique using anatomical surface landmarks (Yu landmark) for L4/L5 interlaminar spinal endoscopic surgery, retrospectively compared with traditional experience-dependent methods. Our findings demonstrate that the new method provides significant improvements in puncture efficiency and safety: it greatly reduces the number of trial punctures and fluoroscopic exposures, reduces radiation exposure by more than half, and significantly improves single-puncture success rates. These technical advantages translate to shorter channel establishment times and slightly better early postoperative pain relief and functional recovery in the landmark group, without any trade-offs in overall surgical effectiveness or increased complications.

Frequent needle repositioning in traditional POSTERIOR INTERLAMINAR SPINE ENDOSCOPY has long been considered a challenge (7). Surgeons, particularly those in the early stages of the learning curve, must rely on iterative fluoroscopic guidance to complete puncture and channel establishment. This can be frustrating and time-consuming—as observed in our traditional group with multiple fluoroscopic images. High fluoroscopic usage not only prolongs operative time but also exposes patients and surgical teams to more ionizing radiation, raising radiation safety concerns (8, 9). Our control group's fluoroscopic count (approximately 23 times) is consistent with literature reports: for example, Ahn et al. and others note that routine POSTERIOR INTERLAMINAR SPINE ENDOSCOPY may involve approximately 20–30 fluoroscopic exposures per case (10). Repeated needle passes through soft tissues may also increase postoperative soreness and risk of inadvertent structural injury. Each additional attempt may irritate or even damage nerve roots, cause muscle hematoma, or tear the annulus fibrosus or ligamentum flavum (11, 12).

Various innovations have been introduced to address this problem of precise puncture positioning. He et al. develop the HE lumbar positioning (HELLO) system, which consists of specialized external positioning devices and puncture guidance frameworks to predetermine trajectory. In a prospective study, this system significantly improves puncture accuracy and reduces fluoroscopic usage. They report that the average number of puncture attempts decreases from approximately 6 times with freehand technique to approximately 1 time with HELLO-guided method, and average fluoroscopic exposures decrease from approximately 25 times to 14 times—these differences are statistically significant (*p* < 0.001). Additionally, their total puncture time and total operative time are shorter in the guided group, with no increase in complication rates (4). Other approaches, such as computer navigation and even robot-assisted positioning, also show similar benefits. Qin et al. demonstrate that fluoroscopy-based navigation systems for POSTERIOR INTERLAMINAR SPINE ENDOSCOPY access can reduce operative time, decrease the number of fluoroscopic images, and shorten the positioning phase compared to conventional techniques (13). Ultrasound-guided puncture and CT-guided systems have also been explored, each aiming to make the puncture step more precise and less radiation-intensive (14). These studies collectively emphasize that technical assistance for positioning can meaningfully improve surgery efficiency and safety.

Our Yu landmark follows this line of thinking but implements it more simply: positioning can be completed using only intraoperative C-arm fluoroscopy and the patient's own anatomical landmarks. Specifically, under anteroposterior fluoroscopy, a vertical line from the midpoint of the L4 inferior articular process controls the medial-lateral direction of the needle path, and a tangent line along the posterior edge of the lamina from the highest point of the L4 spinous process base-lamina junction controls the superior-inferior direction of the needle path, with the intersection marking the ideal needle entry point.

Through orthogonal constraints from these two clear bony landmarks, the initial needle trajectory is precisely defined on the pathway entering the L4/L5 interlaminar “window.” This anatomy-based geometric positioning makes the first needle insertion close to the optimal path, thereby significantly reducing the number of times withdrawal and restart are needed. In contrast, traditional freehand positioning requires surgeons to repeatedly correct based on experience, often averaging 5–6 attempts or more. Our Yu landmark group achieves a single-attempt success rate of over 95%, while the traditional group only achieves approximately 82%, precisely reflecting the significant advantage of the new method in reducing “trial and error.”

Furthermore, the Yu landmark trajectory is anatomically designed to pass through a natural “safe corridor” between the exiting L4 and traversing L5 nerve roots. On axial imaging, this corridor corresponds to the interlaminar window bounded superiorly by the L4 inferior articular process and inferiorly by the L5 lamina. The needle pathway enters this space obliquely, running just above the L5 traversing nerve root and below the L4 exiting nerve root, thereby avoiding any direct contact with neural elements. This anatomical configuration provides a protective buffer zone where the needle tip advances within a region devoid of major neural or vascular structures. As a result, the risk of nerve irritation, dural injury, or vascular bleeding during puncture is greatly minimized. This is similar to the Kambin triangle concept in transforaminal approaches: operating within a certain area below the exit nerve root is considered safe for nerve roots (15). The Yu landmark precisely utilizes this safety corridor, ensuring that the puncture needle always stays away from important neural structures before entering the target area. This explains why our study's Yu group has almost no obvious neurological irritation symptoms while achieving higher puncture success rates. Conversely, traditional freehand needle insertion, due to lack of clear landmarks, may be too medial or lateral under guidance, and slight carelessness may touch nerve roots or the dural sac, increasing the risk of irritation or even injury.

The Yu landmark trajectory is nearly coaxial with the posterior edge of the lamina, with the needle tip “following the bone surface,” being naturally constrained by the lamina and articular processes before entering the interlaminar space. This “bony guardrail effect” on one hand prevents the needle tip from easily deviating from the safe area, avoiding accidental intrusion too deep into the spinal canal; on the other hand, because the needle path advances along the bone surface, the interlaminar space and ligamentum flavum attachment points can be identified under fluoroscopy. When the needle tip reaches the inner edge of the L4 lamina and the origin and insertion of the ligamentum flavum, it indicates that the ideal fenestration position has been reached. Surgeons can accurately perform ligamentum flavum incision (“breaking the yellow”) based on this to establish the interlaminar channel. This strategy of “following bone to find ligamentum flavum” allows us to quickly complete interlaminar fenestration decompression without extensive searching for ligamentum flavum edges (16, 17).

Under endoscopic direct vision, subsequent nucleus pulposus removal or decompression operations are also more comfortable and safe. Studies point out that preserving the deep layer of ligamentum flavum as a barrier during posterior endoscopic decompression can protect neural structures and facilitate drilling and bone decompression. In our method, initial drilling is performed along the inner edge of the lamina, and most bony decompression is completed when the ligamentum flavum is intact. Only after the bone window is formed and the ligamentum flavum boundary is exposed is the ligamentum flavum removed to enter the spinal canal. This “outside-in” stepwise decompression approach is consistent with technical concepts reported in the literature and effectively improves decompression safety.

The Yu landmark technique also has an important advantage in the controllability of decompression range and protection of segmental stability. Because the initial needle path enters close to the medial articular process, we only need to grind away the medial edge of the L4 inferior articular process and a small amount of lamina during reaming to fully expose the dural sac and nerve roots, without excessive bone removal. This means that most of the facet joint is preserved. In this study, we control medial articular process removal to no more than the medial 1/2 of the articular process, preserving at least 50% of the lateral facet joint structure. Such decompression boundaries both ensure adequate decompression of nerve root exit and central canal while avoiding excessive articular process destruction. Biomechanical studies show that removing more than 50% of facet joints significantly increases the risk of segmental instability. Conversely, preserving at least half of the articular process can maintain segmental stability without inducing subsequent intervertebral instability or slippage due to decompression (18–20).

LE-ULBD technique literature also emphasizes that using the medial edge of the pedicle as a landmark to limit decompression range can preserve sufficient articular process structure “without crossing the midline,” thereby reducing iatrogenic instability. Therefore, Yu landmark naturally limits the decompression range within safety thresholds: medial decompression adequately relieves compression, while the lateral half of the articular process is completely preserved, thus balancing decompression effect and spinal stability.

This characteristic may explain why Yu group patients have faster improvement in early postoperative VAS pain and ODI functional scores, because surgery causes less interference with stable structures and soft tissues, resulting in lighter postoperative reactions (21, 22).

The Yu landmark essentially “externalizes” experts' implicit experiential points into clear geometric rules, reducing the technique's subjective dependence and learning curve. Through the “articular process midpoint vertical line” under anteroposterior fluoroscopy and the “spinous process-lamina tangent line” under lateral fluoroscopy, each fluoroscopic exposure provides quantifiable positioning reference.

This single-center retrospective study is subject to selection bias and residual confounding; prospective, ideally randomized, multicenter studies are warranted for further validation. The study cohort is restricted to L4/L5 cases with relatively normal bony anatomy, and its applicability to high-grade spondylolisthesis, severe deformity, scoliosis, or revision surgery remains uncertain. All procedures are performed by experienced surgeons, which may underestimate the potential advantages of the Yu Landmark Method for beginners; future stratified analyses based on surgeon experience are needed. Radiation data are derived from patient machine readings, and actual surgeon exposure levels and long-term occupational risks are not directly assessed.

## Conclusions

The Yu landmark offers a simple and reproducible puncture strategy for L4/L5 posterior interlaminar endoscopic surgery, significantly reducing fluoroscopy, radiation, and channel-establishment time while improving first-pass success. It provides safer, more efficient puncture with comparable long-term outcomes, making it a practical and promotable clinical technique.

## Data Availability

The raw data supporting the conclusions of this article will be made available by the authors, without undue reservation.
